# Use of ginger to control nausea and vomiting caused by chemotherapy in patients with cervical cancer undergoing treatment: An experiment

**DOI:** 10.1097/MD.0000000000029403

**Published:** 2022-06-17

**Authors:** Romeika Lorena Mendes da Silva, Tâmara Taynah Medeiros da Silva, Renata Lima Pessoa, Ayane Cristine Alves Sarmento, Kleyton Santos Medeiros, Daniele Vieira Dantas, Rodrigo Assis Neves Dantas

**Affiliations:** a Research and Innovation Teaching Institute, Liga Contra o Cancer. Natal, RN, Brazil.; b Nursing Graduate Program, Federal University of Rio Grande do Norte, Natal, Brazil.; c Health Sciences Postgraduate Program, Federal University of Rio Grande do Norte (UFRN), Natal, RN, Brazil.; d Department of Nursing, Federal University of Rio Grande do Norte (UFRN), Natal, RN, Brazil.

**Keywords:** ginger, antineoplastic agents, vomiting, nausea

## Abstract

**Introduction:**

Uterine cervix tumors have an invasive nature, with the capacity to proliferate to surrounding organs such as the vagina, bladder, and rectum, as well as the capacity for dissemination and involvement of structures distant from its place of origin. According to the International Federation of Gynecology and Obstetrics, patients with stages IB I, IB I microscopic (small dimension <4 cm) are indicated for radiotherapy or adjuvant chemoradiotherapy with cisplatin (40 mg/m^2^). However, cisplatin has side effects such as hematological implications (anemia, neutropenia, and thrombocytopenia), gastrointestinal disorders (nausea, vomiting, diarrhea, constipation), and fatigue. *Zingiber officinale* contains bioactive compounds that act on pregnancy and postoperative nausea, chemotherapy-induced nausea and vomiting, and also in the management of fatigue, myalgia, and insomnia. This study aimed to evaluate the effects of ginger on chemotherapy-induced nausea and vomiting in patients with cervical cancer undergoing treatment with cisplatin and radiotherapy.

**Methods and analyses:**

A randomized intervention clinical and controlled trial with a triple-blind design is described, comparing the effects of institutional antiemetic therapy alone, as well as in combination with 2 different ginger concentrations.

**Ethics and dissemination:**

Due to the nature of the study, we obtained approval from the Division Ethics Committee of Liga Contra o Câncer. All participants signed an informed consent form prior to randomization. The results of this study will be published in peer-reviewed journals. The data collected will also be available in a public repository of data.

**Trial registration number:**

This study is registered in the Brazilian Registry of Clinical Trials under number RBR-47yx6p9. This study was approved by the Division Ethics Committee of Liga Contra o Câncer under CAAE 40602320.0.0000.5293.

**STRENGTHS AND LIMITATIONS OF THIS STUDY**

- This is the first randomized controlled trial comparing ginger doses with placebo doses for controlling chemotherapy-induced nausea and vomiting (CINV).
- Inclusion criteria allow for homogeneity of subjects and less risk of bias.
- Blinding of assessors and standardization of protocols enhance this trial's internal validity.
- The primary outcome includes controlling nausea and vomiting or common terminology criteria for adverse events (CTCAE) (gastrointestinal disorders - nausea/vomiting).

## 1 Introduction

Cancer is a leading cause of morbidity and mortality worldwide, with 19.2 million new cases reported annually.^[1]^ In 2020, an estimated 9.9 million deaths occurred due to cancer, and the estimated prevalence over the past 5 years involved 50.1 million people. Among the malignant neoplasms, cervical cancer predominantly affects females. It is the fourth most frequent cancer worldwide with a prevalence of 1.5 million cases, and an incidence of 605,000 new cases worldwide.^[1]^ The cervix tumor has an invasive feature with the capacity to proliferate to surrounding organs such as the vagina, bladder, and rectum, as well as dissemination and involvement of structures distant from its place of origin.^[1,2]^ This neoplasm is mainly caused by human papillomavirus (HPV) infection, early sexual activity with multiple partners, smoking, and prolonged use of oral contraceptives.^[3]^

The treatment of cervical cancer has 3 aspects: surgery, chemotherapy, and radiotherapy, which can be performed alone or in combination. According to the International Federation of Gynecology and Obstetrics staging system, patients with stages IB I, IB I microscopic (small dimension <4 cm), are indicated for radiotherapy (RDT) or adjuvant chemoradiotherapy with cisplatin (40 mg/m^2^). Administration of weekly cisplatin with radiotherapy shows superior results compared to isolated radiotherapy, and promotes less toxicity than in conjunction with other drugs.^[4]^ However, cisplatin has side effects, such as hematological abnormalities (anemia, neutropenia, and thrombocytopenia), gastrointestinal disorders (nausea, vomiting, diarrhea, constipation), and fatigue.^[1,5,6]^

Chemotherapy-Induced Nausea and Vomiting (CINV) are considered severe side effects that most commonly occur during cancer treatment, including treatment with cisplatin.^[7–9]^ The management of these side effects becomes difficult to control owing to the multiple central and peripheral neural stimuli caused by the drugs.^[4,10,11]^

Repetitive episodes of nausea and/or emesis may result in decreased food intake causing loss of nutrients and water, metabolic disorders due to gastric losses, anorexia, fatigue, and increased risk of acute renal failure. In addition, psychosocial aspects such mental deterioration and acceptance of body self-image can compromise treatment adherence and cause abandonment.^[7,12]^

As *Zingiber officinale* contains bioactive compounds, such as gingerols, shogaols, zingiberene, zingerone and paradol (gingerol and shogaol) it is affective against pregnancy and postoperative nausea such as CINV, as well as in the management of fatigue, myalgia, and insomnia.^[13–17]^ However, these active components directly influence the antral motility, optimizing gastric emptying and intestinal peristalsis. They also affect the central nervous system, thus modulating serotoninergic pathways of 5-HT^3^ receptors, as well as the drugs mentioned above.^[16]^ Experimental studies confirm the safety of the root and its effectiveness in the treatment and prevention of secondary symptoms of chemotherapy.^[18,19]^

Therefore, this study aims to evaluate the effects of ginger on CINV in patients with cervical cancer undergoing treatment with cisplatin and radiotherapy.

### 1.1 Objectives

To evaluate the effects of ginger on CINV in patients with cervical cancer treated with cisplatin and radiotherapy.

## 2 Methods and analysis

Our protocol adheres to the Standard Protocol Items for Randomized Trials (SPIRIT) and Consolidated Standards of Reporting Trials (CONSORT) statements.^[20]^

### 2.1 Trial design

This is a protocol of a randomized clinical and controlled intervention trial with a triple-blind design, which compares the effects of institutional antiemetic therapy alone as well as with the use of 2 different ginger concentrations.

### 2.2 Population

The treatments will be carried out in a reference center for cancer care in Brazil. Recruitment of participants is ongoing at a referral center for cancer treatment. After reading and signing the informed consent form, patients who meet the eligibility criteria will be included in the research.

### 2.3 Eligibility criteria and recruitment

**Figure 1:**
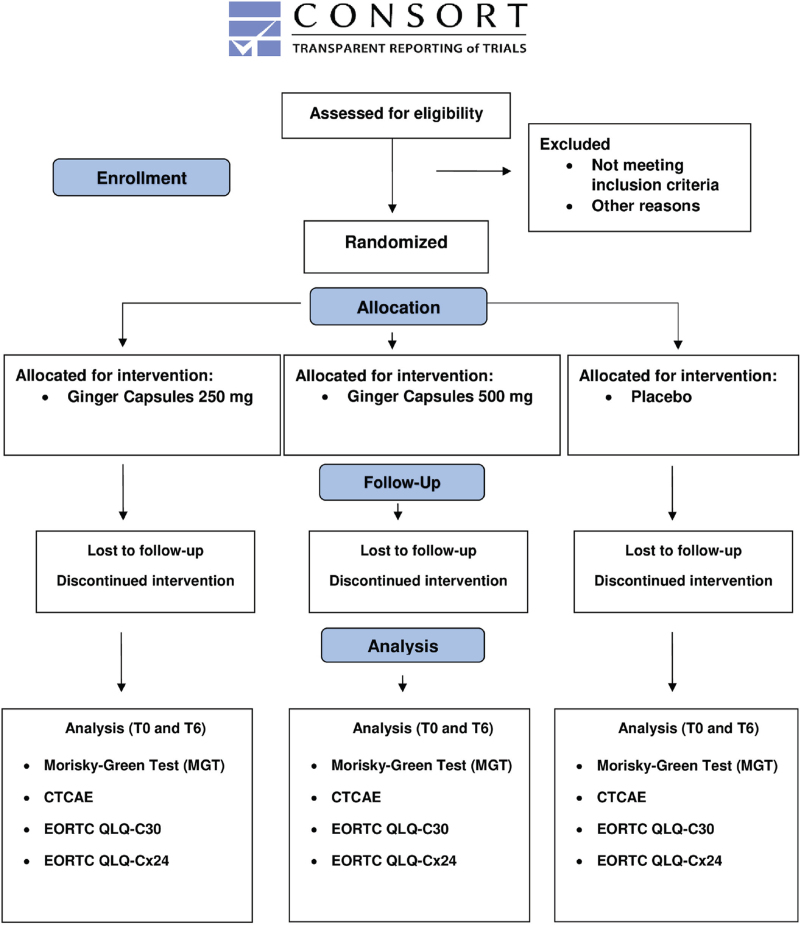
CONSORT 2010 flow diagram.

The study will include patients over 18 years of age, who were diagnosed with cancer of the uterine cervix on histological confirmation. Furthermore, they were indicated for treatment with cisplatin 40 mg/m^2^ associated with radiotherapy and had capsule swallowing capacity. Figure 1 shows the study flow.

### 2.4 Interventions

The control group will swallow capsules containing 500 mg of placebo (77.80% pharmaceutical starch + 10.10% pharmaceutical talc + 10.10% microcrystalline cellulose + 1% magnesium stearate + magnesium dioxide 1% colloidal silicon, contained in white/blue gelatin capsules of size No. 00). Placebo capsules will be swallowed with approximately 250 ml of water every 12 hours, totaling to 1 g per day.

Experimental group 1 will be composed of patients who will receive capsules containing 250 mg of the ginger extract contained in white/blue gelatin capsules No. 00. The capsules will be swallowed with approximately 250 ml of water every 12 hours, totaling to 500 mg per day. The ginger used will be dehydrated and crushed into a powder form without any chemical additives.

Finally, experimental group 2 will receive capsules containing 500 mg of the ginger extract contained in white/blue gelatin capsules No. 00 and will be swallowed with an average of 250 ml of water every 12 hours totaling to 1 g per day. The ginger will be dehydrated and crushed into a powder form without any chemical additives.

### 2.5 Questionnaire

In the first query, the participants will answer a standardized questionnaire with information on demographic characteristics, including age, marital status, profession, schooling, socioeconomic classification, and risk factors (alcoholism, smoking, HPV, use of oral contraceptives, and whether Pap test was regularly performed).

### 2.6 Outcomes

#### 2.6.1 Primary outcome

Seven relevant time points will be considered for evaluating the results based on the chemotherapy protocol of cisplatin with radiotherapy: baseline (P0), 7 days after the first dose of medication (P1), on every consecutive 7th day during a break in treatment (P2, P3, P4, P5, and P6), and 15 days after the last dose (P7).

The primary outcome includes controlling nausea and vomiting as evaluated using the Common Terminology Criteria for Adverse Events or CTCAE (gastrointestinal disorders-nausea/vomiting).^[21]^ The CTCAE for nausea (a disorder characterized by a queasy sensation and/or the urge to vomit) will be classified as Grade 1 (loss of appetite without alteration in eating habits), Grade 2 (oral intake decreased without significant weight loss, dehydration, or malnutrition), or Grade 3 (inadequate oral caloric or fluid intake; tube feeding, parenteral nutrition, or hospitalization indicated). Vomiting (a disorder characterized by the reflexive act of ejecting the stomach contents through the mouth) will be classified as Grade 1 (intervention not indicated), Grade 2 (outpatient IV hydration: medical intervention indicated), Grade 3 (tube feeding, parenteral nutrition, or hospitalization indicated), Grade 4 (life-threatening consequences), or Grade 5 (death) (Table 1).

**Table 1:** Outcome measurements.

Outcome measurement	Explanation	Time points for assessment
CTCAE	Common terminology criteria for adverse events	T1, T2, T3, T4, T5, and T6
EORTC QLQ C30	European Organization for Research and Treatment of Cancer Quality of Life Questionnaire Core 30	T1, T2, T3, T4, T5, and T6
EORTC QLQ Cx24	EORTC quality of life cervical cancer module	T1, T2, T3, T4, T5, and T6
MGT	Morisky-Green Test to evaluate medication adherence	T1, T2, T3, T4, T5, and T6

#### 2.6.2 Secondary outcome

Secondary outcomes will be quality of life module of the European Organization for Research and Treatment of Cancer and Cervical Cancer- Specific Quality of Life Questionnaire, and medication adherence as evaluated using the Morisky-Green Test.^[22–25]^

The European Organization for Research and Treatment of Cancer is an internationally validated and widely used cancer-specific health related quality of life (HRQoL) instrument. It contains 5 scales of functioning (physical, social, role, cognitive, and emotional functioning), 8 symptom scales (fatigue, nausea/vomiting, pain, dyspnea, sleep disturbances, appetite loss, constipation, and diarrhea), and evaluations of financial impact and overall quality of life. Raw scores are linearly converted to a scaled score in a range between 0 and 100.^[22]^ For the functioning scales and global QOL, higher scores indicate better functioning; for the symptom scales, higher scores indicate higher symptom burden. Furthermore, the cervical cancer-specific EORTC QLQ-CX24 consists of 5 multi-item scales on clinically distinct dimensions (sexual functioning, body image, gastrointestinal, urologic, and vaginal symptoms) and several single-item measures.^[23]^

To evaluate medication adherence, we will use the version of the Morisky-Green Test which includes the following questions: Do you sometimes have difficulty remembering to take your medication? Do you sometimes not pay attention to taking your medication? When you feel better, do you sometimes stop taking your medication? Sometimes, if you feel worse after taking medication, do you stop taking it?^[24]^

The evaluation of the results is described in Table 2. All possible adverse effects will be recorded and qualified during the treatment period using questionnaires developed for this protocol. Any adverse events will be reported and discussed in the Results section of the manuscript. Any breach of confidentiality, study protocol, or adverse events attributable to this study will be reported to the research ethics committees.

**Table 2:** Schedule of enrollment, interventions, assessments and data collection.

Study period								
	Enrollment/baseline	Intervention						Follow-up
Time point	T0	T1	T2	T3	T4	T5	T6	15 d
Enrollment	X							
Eligibility screen	X							
Informed consent	X							
Randomization	X							
Interventions								
Ginger capsules 250 mg		X	X	X	X	X	X	
Ginger capsules 250 mg		X	X	X	X	X	X	
Placebo		X	X	X	X	X	X	
Assessments								
General condition								X

### 2.7 Screening

After a nursing consultation, the elected patient will fill a registration form containing patient identification data and clinical and demographic information. Next, a pharmacist will dispense a bottle of capsules corresponding to the participant's previously determined group allocation.

### 2.8 Follow-up

Follow-up data including a general condition assessment will be recorded during the follow-up period corresponding to the multiple time points. The details are presented in Table 2.

Each patient will remain in the study for 7 weeks or until the end of chemotherapy treatment if the chemotherapy period is scheduled to end before the 7-week intervention. Recruitment and data collection will take place at the following times:

**Period 1:** Recruitment (Day 0) at the time of chemotherapy initiation.
**Period 2:** Intervention (Weeks 16), data will be recorded every 7 days or at the time of each new weekly chemotherapy dose.
**Period 3:** Time after intervention (at least 7 but within 15 days after completion of treatment), follow-up for long-term results.
The last meeting between the researcher and patient can take place via phone call or in-person, as it will be at least 1 week after the last infusion of the antineoplastic therapy.
At the weekly chemotherapy session, the principal researcher will check the number of remaining capsules to assess the adherence to study protocol. In all, will be 7 in-person possibilities for data collection. If no return is established, telephone contact will be attempted in the last week.

### 2.9 Sample size

The sample size calculation will be established using G Power Software version 3.1.9.2 (https://www.psychologie.hhu.de/arbeitsgruppen/allgemeine-psychologie-und-arbeitspsychologie/gpower), considering a Cohen effect size of 0.40, a test power of 0.80, and a significance level of 5% (*P* value <.05).^[26]^ The sample is estimated to be of 39 patients with a potential addition of 20%, totaling 48 participants, which will be equally allocated (1:1:1) into 3 groups of 16 patients each (the ginger capsules 250 mg group, ginger capsules 500 gm group, and placebo group).

### 2.10 Randomization and allocation concealment

On providing informed consent, eligible participants will be randomized by the Software Research Randomizer program using block design (1:1:1; experimental group 1, experimental group 2, or control group). Patients will be randomized into 3 intervention groups.

To ensure blind allocation, an off-site randomization schedule will be followed. This schedule will be prepared by a researcher at the Institute of Education, Research and Innovation (Liga Contra o Câncer), who will have no contact with any participants throughout the trial and will not be involved in the recruitment, screening, assessment, enrollment, or treatment process. To enroll a participant, the primary researcher will email the consenting participant's name to the researcher at the LIGA. These details will be entered into the allocation spreadsheet, and the corresponding treatment allocation and participant identification number will be emailed to the researcher.

### 2.11 Blinding

Research participants, group researchers, and individuals who assess the outcomes will not have access to the details of group assignments. This ensures that bias for or against the tested treatment is unlikely to occur.

### 2.12 Data management

Initially, an experienced team member (RLMS) will be trained to collect data according to the study protocol. The qualification of the researcher is essential to ensure the quality of clinical trials. Data collection will be carried out through the REDCap website. Data management included baseline characteristics (demography, lifestyle, comorbidities, history of HPV treatment, history of long-term use of oral contraceptives, tumor characteristics, International Federation of Gynaecology and Obstetrics staging, and inclusion and exclusion criteria), potential confounds, and outcomes as per the QOL questionnaire. Participants who withdraw from our study will be followed up with, and data will be analyzed according to the intention-to-treat principle. All randomized participants will be followed up with for 2 months after randomization.

### 2.13 Data extraction and statistical analysis

For the analysis of quantitative data, repeated measures ANOVA based on the linear mixed effects model will be used. A binary variable will also be created to indicate the occurrence of nausea in the 24 hours after the infusion in at least 1 of the 6 weeks. In this case, a logistic regression model will be used and the odds ratio of occurrence of nausea will be calculated for the 3 groups. Data presented in the text and tables will be reported as means and SDs, mean, absolute values and percentages (%). Statistical significance will be set at *P* <.05. The software that will be used is the R Project for Statistical Computing for Windows, VR version 4.0.2.

### 2.14 Patient and public involvement

All the patient information will be anonymously analyzed and processed. The results of our trial will be disseminated to the participating patients through a letter after publication.

## 3 Discussion

This protocol entails a randomized trial that will determine the effects of antiemetic therapy associated or isolated with ginger on CINV is described. The strengths of this research include a triple-blind study method, which avoids ethical deviations and placebo-controlled randomization, and the ability to evaluate several simultaneous clinical outcomes. The limitations include the lack of adherence to the use of the proposed drugs, and lack of follow-up after the treatment. Subsequently, reduction of neutrophils beyond the minimum limit (neutropenia), although not a factor disconnection from the search, will delay the progress of the search and changes to the chemotherapy protocol may be required to ensure project completion.

Few studies with the same methodological design show positive results associated with the use of ginger. A randomized clinical study revealed that ginger supplementation at doses 0.5 g and 1.0 g significantly relieves the severity of acute nausea (within 24 hours).^[27]^ In addition, it delays CINV when associated with ondansetron and dexamethasone in a chemotherapy regimen with high emetogenic power.^[17]^

However, an experimental study that compared the effectiveness of ginger and chamomile in the management of cancer treatment induced gastrointestinal effects revealed that both herbal medicines reduce the frequency of vomiting with no significant difference between the 2 groups; however, ginger offered a notable reduction in the frequency of nausea.^[28,29]^ Furthermore, a clinical trial revealed that ginger can not only control nausea and vomiting, but can also enhance the quality of life of chemotherapy patients by reducing fatigue by up to 80%.^[30,31]^

## 4 Ethics and dissemination

All procedures performed in this study involving human subjects will be conducted following the ethical standards of the 1964 Declaration of Helsinki and its subsequent amendments, the Madrid Declaration of the World Psychiatric Association, and the requirements established for manuscripts submitted to biomedical journals or ethical standards of good practice clinics. This trial was approved by the Human Research Ethics Committee of the North Riograndense Against Cancer, under the number CAAE 40602320.0.0000.5293 (approval date: February 3, 2021) and was registered in the Brazilian Registry of Clinical Trials under the number RBR-47yx6p9. Before registration of the trial participants, data confidentiality will be guaranteed through data anonymization.

## Author contributions

RLMS, TTMS, RLP, and DVD were involved in drafting the study protocol. TTMS and RLP were involved in the statistical planning and drafting of the study protocol. KSM and RAND were involved in drafting and revising the study protocol. RAND and KSM developed the idea for this trial and were involved in drafting and revising the study protocol. RLMS and TTMS conceived and designed the concept for this trial, was involved in drafting and revising the study protocol, and was the trial's principal investigator. All authors will be involved in data acquisition and approval of the final version of the manuscript.

**Conceptualization:** Ayane Alves Sarmento, Daniele Dantas, Kleyton Medeiros, Renata Pessoa, Rodrigo Neves Dantas, Romeika Mendes da Silva, Tâmara Medeiros da Silva.

**Investigation:** Romeika Mendes da Silva.

**Methodology:** Ayane Alves Sarmento, Kleyton Medeiros, Renata Pessoa, Rodrigo Neves Dantas, Romeika Mendes da Silva, Tâmara Medeiros da Silva.

**Project administration:** Renata Pessoa, Rodrigo Neves Dantas.

**Supervision:** Kleyton Medeiros, Rodrigo Neves Dantas.

**Validation:** Romeika Mendes da Silva.

**Visualization:** Kleyton Medeiros.

**Writing – original draft:** Ayane Alves Sarmento, Daniele Dantas, Kleyton Medeiros, Renata Pessoa, Rodrigo Neves Dantas, Romeika Mendes da Silva, Tâmara Medeiros da Silva.

**Writing – review & editing:** Daniele Dantas, Kleyton Medeiros, Renata Pessoa, Rodrigo Neves Dantas.

## References

[1] Cancer Today [Internet]. [cited 2021 Oct 28]. Available at: https://gco.iarc.fr/today/online-analysis-pie?v=2020&mode=cancer&mode_population=continents&population=900&populations=900&key=total&sex=0&cancer=39&type=0&statistic=5&prevalence=0&population_group=0&ages_group%5B%5D=0&ages_group%5B%5D=17&nb_items=7&group_cancer=1&include_nmsc=1&include_nmsc_other=1&half_pie=0&donut=0.

[2] Yarbro C, Wujcik D, Gobel B. Cancer Nursing Principles and Practice [Internet]. 8th ed. Yarbro CH, Wujcik D, Gobel BH, editors. 2018 [cited 2021 Oct 29]. Available at: https://books.google.com.br/books?id=mGt7jgEACAAJ&printsec=frontcover&hl=pt-BR&source=gbs_ge_summary_r&cad=0#v=onepage&q&f=true.

[3] WHO. Human papillomavirus (HPV) and cervical cancer [Internet]. 2019 [cited 2021 Oct 20]. Available at: https://www.who.int/news-room/fact-sheets/detail/human-papillomavirus-(hpv)-and-cervical-cancer.

[4] Marx W Ried K McCarthy AL. Ginger—mechanism of action in chemotherapy-induced nausea and vomiting: a review. Crit Rev Food Sci Nutr 2017;57:141–6. Available from: Ginger—mechanism of action in chemotherapy-induced nausea and vomiting: a review. 25848702 10.1080/10408398.2013.865590

[5] Chuang LT Feldman S Nakisige C Temin S Berek JS. Management and care of women with invasive cervical cancer: ASCO resource-stratified clinical practice guideline. J Clin Oncol 2016;34:3354–5. 27382101 10.1200/JCO.2016.68.3789

[6] UpToDate. Cisplatina: informações sobre medicamentos [Internet]. 2020 [cited 2021 Oct 21]. Available at: https://www.uptodate.com/contents/cisplatin-drug-information?search=cisplatina&source=panel_search_result&selectedTitle=1∼148&usage_type=panel&kp_tab=drug_general&display_rank=1#F151928.

[7] NCBI. Nausea and Vomiting Related to Cancer Treatment (PDQ®) - PDQ Cancer Information Summaries - NCBI Bookshelf [Internet]. 2020 [cited 2021 Oct 20]. p. 1–70. Available at: https://www.ncbi.nlm.nih.gov/books/NBK66056/.

[8] Longstreth GF. Approach to the adult with nausea and vomiting [Internet]. 2020 [cited 2021 Oct 25]. Available at: https://www.uptodate.com/contents/approach-to-the-adult-with-nausea-and-vomiting?search=vomiting&source=search_result&selectedTitle=1∼150&usage_type=default&display_rank=1.

[9] MOC. CISPLATINA - Manual de Oncologia Clínica do Brasil [Internet]. 2020 [cited 2021 Oct 20]. Available at: https://mocbrasil.com/moc-drogas/agentes-oncologicos/1-agentes-oncologicos/cisplatina/.

[10] MASCC, ESMO. Guideline update for the prevention of chemotherapy- and radiotherapy-induced nausea and vomiting and of nausea and vomiting in advanced cancer patients. Ann Oncol 2016;27:v119–33. 27664248 10.1093/annonc/mdw270

[11] NCCN. Guidelines Version 2. 2020 Antiemesis. [Internet] 2020. [cited 2021 Oct 27]. Available at: https://www.nccn.org/professionals/physician_gls/pdf/antiemesis.pdf.

[12] Heckroth M Luckett RT Moser C Parajuli D Abell TL. Nausea and vomiting in 2021: a comprehensive update. J Clin Gastroenterol 2021;55:279–99. 33471485 10.1097/MCG.0000000000001485 PMC7933092

[13] Saad M de Medeiros R. Uso do gengibre para controle de náusea e vômito. Educ Contin Saúde einstein 2013;11:29–30.

[14] Li X Qin Y Liu W Zhou XY Li YN Wang LY. Efficacy of ginger in ameliorating acute and delayed chemotherapy-induced nausea and vomiting among patients with lung cancer receiving cisplatin-based regimens: a randomized controlled trial. Integr Cancer Ther 2018;17:747–54. 29417850 10.1177/1534735417753541 PMC6142108

[15] Soares MB Stringhini MLF Freitas AS. Efeito do Gengibre (*Zingiber officinale*) na qualidade de vida do paciente em quimioterapia. Braz J Dev 2019;5:18988–9002.

[16] Borges DO Freitas KABS Minicucci EM Popim RC. Benefits of ginger in the control of chemotherapy-induced nausea and vomiting Rev Bras Enferm [Internet] 2020;73:e20180903. 10.1590/0034-7167-2018-0903 32236378

[17] Nicácio GLS, Moura SC, De J v, Costa J, Sena CR, De T, et al. Breve revisão sobre as propriedades fitoterápicas do *Zingiber officinale* roscoe-o gengibre brief review on phytotherapy properties of *Zingiber officinale* roscoe-ginger [Internet]. Vol. 7. 2018. Available at: http://periodicos.pucminas.br/index.php/sinapsemultipla.

[18] Pillai AK Sharma KK Gupta YK Bakhshi S. Anti-emetic effect of ginger powder versus placebo as an add-on therapy in children and young adults receiving high emetogenic chemotherapy. Pediatric Blood Cancer 2011;56:234–8. 20842754 10.1002/pbc.22778

[19] Ryan JL Heckler CE Roscoe JA Dakhil SR Kirshner J Flynn PJ. Ginger (*Zingiber officinale*) reduces acute chemotherapy-induced nausea: a URCC CCOP study of 576 patients. Support Care Cancer 2012;20:1479–89. 21818642 10.1007/s00520-011-1236-3 PMC3361530

[20] Supportive Care in Cancer. [Internet] 2012;20:1479-89. 2010 [cited 2021 Oct 29]. Available at: https://pubmed.ncbi.nlm.nih.gov/21818642/.

[21] Consort. Consort - Welcome to the CONSORT Website [Internet]. 2010 [cited 2021 Oct 29]. Available at: http://www.consort-statement.org/.

[22] Cancer Institute N. Common Terminology Criteria for Adverse Events (CTCAE) v5.0 [Internet]. 2017 [cited 2021 Oct 29]. Available at: https://www.meddra.org/.

[23] Gundy CM Fayers PM Groenvold M. Comparing higher order models for the EORTC QLQ-C30. Qual Life Res 2012;21:1607–17. 22187352 10.1007/s11136-011-0082-6 PMC3472059

[24] Greimel ER Vlasic KK Waldenstrom AC. On behalf of the European Organisation for Research and Treatment of Cancer (EORTC) Quality of Life Group. The European Organisation for Research and Treatment of Cancer (EORTC) Quality-of-Life Question-naire cervical cancer module, EORTC QLQ-CX24. Cancer 2006;107:1812–22. 16977652 10.1002/cncr.22217

[25] Morisky DE Green LW Levine DM. Validade concorrente e preditiva de uma medida autorrelatada de adesão à medicação. Med Care 1986;24:67–74. 3945130 10.1097/00005650-198601000-00007

[26] Cohen J. Statistical Power Analysis for the Behavioral Sciences Second Edition. 1988.

[27] Ryan JL Heckler CE Roscoe JA. Ginger (*Zingiber officinale*) reduces acute chemotherapy-induced nausea: a URCC CCOP study of 576 patients. Support Care Cancer 2012;20:1479–89. 21818642 10.1007/s00520-011-1236-3 PMC3361530

[28] Fitriyanti D Sulung R. INTERNATIONAL COLUMN effectiveness of ginger to overcome nausea and vomiting caused by chemotherapy in breast cancer patients. Can Oncol Nurs J 2020;30:03–5. 10.5737/2368807630135 PMC7585706 33118988

[29] Sanaati F Najaf S Kashaninia Z Sadeghi M. Effect of ginger and chamomile on nausea and vomiting caused by chemotherapy in Iranian women with breast cancer. Asian Pac J Cancer Prev 2016;[cited 2021 Oct 29];4125-9. 27644672

[30] Crichton M Marshall S Marx W McCarthy AL Isenring E. Efficacy of ginger (*Zingiber officinale*) in ameliorating chemotherapy-induced nausea and vomiting and chemotherapy-related outcomes: a systematic review update and meta-analysis. J Acad Nutri Diet 2019;119:2055–68. 10.1016/j.jand.2019.06.009 31519467

[31] Marx W McCarthy AL Ried K. The effect of a standardized ginger extract on chemotherapy-induced nausea-related quality of life in patients undergoing moderately or highly emetogenic chemotherapy: a double blind, randomized, placebo controlled trial. Nutrients 2017;9:10.3390/nu9080867 PMC5579660 28805667

